# Editorial: The Sensation-Cognition Interface: Impact of Early Sensory Experiences on Cognition

**DOI:** 10.3389/fpsyg.2017.01742

**Published:** 2017-10-06

**Authors:** Matthew W. G. Dye, Olivier Pascalis

**Affiliations:** ^1^Department of Liberal Studies, Rochester Institute of Technology, Rochester, NY, United States; ^2^CNRS, Université Grenoble Alpes, Saint-Martin-d'Hères, France

**Keywords:** blindness, language deprivation, plasticity, cognitive development, spatial localization, auditory perception, visual perception, deafness

The fourteen articles in this special topic are linked by their consideration of how various kinds of experiential deprivation can affect cognitive development. Reflecting recent increased interest in multisensory processing, most submissions look at how the absence of one sensory modality influences processing in another. Codina et al. studied perceptual changes in the peripheral visual field induced by deafness and/or sign language. They compared performance in the central and peripheral vision of deaf signers, hearing signers and hearing non-signers in a forced choice task. Deaf participants performed better than the other groups for peripherally presented stimuli, suggesting that sign language acquisition alone does not change peripheral vision in the way that deafness does. Samar and Berger investigate the hypothesis of reallocation of attentional resources from the central to the peripheral visual field in the deaf, using a spatial attention paradigm requiring target localization. Comparing deaf participants with or without cochlear implants (CIs) with hearing participants, they surprisingly observed that deaf with no CIs show a reduced central attentional capacity which was not associated with enhanced peripheral attention. In the perception of faces, a left visual field bias typically exists which suggests a right hemisphere specialization. This bias is already observed in 5-year-old hearing children. Dole et al. found a reduced left visual field bias in deaf adults compare to hearing peers when using chimeric faces. This result was associated with increased looking time toward the mouth for deaf participants. Early profound deafness is therefore associated with differences in face scanning and could induce a change in hemispheric specialization possibly linked to speech reading. Pimperton et al. investigated whether deaf adults with CIs showed significantly better scores on a test of speechreading than hearing, hypothesizing that the age of implantation might influence this ability. They found that the deaf with CIs were better speechreaders than the hearing, with a significant positive correlation between age at implantation and speechreading performance: earlier implantation was associated with poorer speechreading scores. Aparicio et al. investigated the neural substrate of speech reading and speech with cued speech (CS) recruiting deaf individuals, CS users, and individuals with typical hearing. Their study explores the neural similarities and differences in processing oral language delivered in a visuo-manual or in an audio-visual modality. They found a common, amodal neural basis for the perception of both audiovisual speech and CS, but clear differences were observed in the posterior parts of the superior temporal sulcus for auditory and speechread information in audiovisual speech, and in the occipitotemporal junction for CS. Corina et al. recorded auditory and visual evoked potentials in hearing children and deaf children with CIs. They reported an atypical auditory P1 in the deaf children, even with early implantation and significant auditory experience, which they interpret as potentially reflecting an aberrant maturation of cortical function early in development. They found no differences in the visual evoked potentials. When analyzing the relationship between auditory P1 and visual N1 within participants, they found a significant correlation in the hearing children not in deaf children with CIs.

In addition to these studies of deaf children and adults, several contributions looked at cognition in blind children and adults. Cappagli et al. looked at auditory spatial localization in preschool children who were congenitally blind or had low vision. Their results suggest that some early visual experience is required for the development of domain-general spatial cognition. Gori et al. report a study that required early blind, late blind and sighted adults to report the shape made by a moving sound source and then to replicate that movement in a locomotion task. They report that early blind, but not late blind, individuals struggled to identify the auditory motion paths and, even when they could correctly do so, their ability to reproduce those shapes in the locomotion task was error prone. Tonelli et al. asked whether vision is necessary for the calibration of auditory space, or whether the same function can be subserved by touch. In blindfolded adults with typical vision, they report that haptic exploration of a 3D model of a room, accompanied by ambulatory exploration of the room while blindfolded, resulted in improved bisection of an auditory line. Subsequent visual exploration did not improve bisection any further. Finally, two reviews explore different aspects of cognition in blind individuals. Voss reviews the literature on auditory spatial cognition in the blind and suggests that data must be interpreted in terms of task demands. In particular, findings differ depending upon whether testing occurs in the horizontal or vertical plane, whether depth judgments are absolute or relative, and whether tasks are performed mono- or binaurally. He suggests that the specificity of effects likely represents the use of different auditory spectral cues. Martin et al. point out the importance of considering etiology in their review of ocular versus cortical/cerebral visual impairment (CVI). They point out that CVI is now a highly prevalent cause of early childhood blindness in developed countries, and that it is associated with a range of attentional and visual dysfunctions that mean rehabilitation strategies designed for those with ocular deficits are ineffective for individuals with CVI.

Together, these eleven articles on sensory deprivation—deafness and blindness—reveal some striking theoretical and practical parallels. The *cross-sensory calibration hypothesis* of Gori et al. (2012) bears a remarkable similarity to the *auditory scaffolding hypothesis* proposed by Conway et al. (2009), and provides a potential unifying framework for understanding how a lack on input in one sensory modality affects development in another [see also the *intersensory redundancy hypothesis* of Bahrick and Lickliter (2000)]. There are also important methodological considerations that arise from both literatures. Voss points out that early and late blind individuals may make use of different auditory spectral cues, and may make use of different reference frames. In addition, he also notes that transient visual deprivation (such as by blindfolding) results in effects not observed in early blind individuals. In the same way, whether or not a deaf child has a CI (and the age at which they receive one) may also results in different patterns of compensatory change, and the work of Bross et al. established that transient auditory deprivation results in visual changes not observed in those born profoundly deaf (Bross et al., 1980). Last of all, Martin et al.'s review of ocular versus cerebral etiologies for visual impairment mirrors discussion in the field of deafness about carefully considering the etiology of hearing loss in deaf individuals (Bavelier et al., 2006).

The last three contributions to this special topic reflect the effects of different kinds of deprivation on cognition—linguistic and social deprivation. Gagne and Coppola ask whether observing social interactions is sufficient to support social cognition in deaf adults who have not acquired conventional language. They report that these Homesigners performed well on simple visual perspective taking tasks. However, they struggled on false belief tasks, as did unschooled signers and speakers of conventional languages. The authors suggest that language may be important for the development of theory of mind, but that it is not sufficient. Henner et al. examined how the age of entry to a signing school for the deaf (and, by proxy, age of sign language exposure) affects the ability to make syntactic judgments and perform an analogical reasoning task. Their data suggest that earlier entry into signing schools for the deaf is, in these children, associated with better ASL syntactic knowledge and improved analogical reasoning skills. Finally, Tibu et al. analyzed data from the Bucharest Early Intervention Project, looking at the effects of institutionalization on the development of ADHD symptoms. They report an analysis to show that the effect of social and communicative deprivation on the emergence of ADHD symptoms at age 12 years is mediated by impaired working memory. These studies show that it is not only sensory experience that shapes cognitive abilities; social and linguistic interaction also play an important role in the development of a child's ability to think and reason. Further research is needed for a better holistic understanding of how sensory, linguistic and social interactions are interrelated and work together to shape and dictate the direction of cognitive development.

## Author contributions

All authors listed have made a substantial, direct and intellectual contribution to the work, and approved it for publication.

### Conflict of interest statement

The authors declare that the research was conducted in the absence of any commercial or financial relationships that could be construed as a potential conflict of interest.
